# 

**Published:** 1870-05

**Authors:** 


*CONTENTS:*

ORIGINAL COMMUNICATIONS:

ART. I. Puerperal Fever in Cook Co. Hospital .......... 193
" II. Incised and punctured wounds of Joints. By I. N. Danforth, M. D. .......... 196
" III. A Case of Aneurism of the Orbit. By E. Powell, M.D. .. 199
" IV. Cerebro-Spinal Meningitis. By Thos. C. Murphy, M.D.... 202
" V. Case of the Dislocation of the Humerus. By T. A. Montgomery .......... 204
" VI. Akephaloid Monster. By N. B. Larsh, M.D., .......... 205

EPISTOLARY:

Letter to the Editor. By "Viator."... 211

ORIGINAL TRANSLATIONS:

ART. I. The Influence of Electrical Currents upon the Nervous System. By Mm. Legros and Oninus........ 211

FOREIGN CORRESPONDENCE:

Letter from A. W. Bosworth, Paris... 222

EDITOR'S BOOK TABLE .......... 223

EDITORIAL:

Ubique Gentium? .......... 233
Albany Medical College .......... 236
Medical Gossip .......... 237

OBITUARY: Death of Jno. Murphy, M.D. 241

ILLINOIS STATE MEDICAL SOCIETY...... 242

LOOT. .......... 243

